# Empirical evaluation of the spatial scale and detection process of camera trap surveys

**DOI:** 10.1186/s40462-021-00277-3

**Published:** 2021-08-14

**Authors:** Roland Kays, Allison Hody, David S. Jachowski, Arielle W. Parsons

**Affiliations:** 1 Department of Forestry and Environmental Resources, North Carolina State University, 2800 Faucette Drive, Raleigh, NC USA; 2 North Carolina Museum of Natural Sciences, 11 West Jones Street, Raleigh, NC USA; 3 Department of Forestry and Environmental Conservation, Clemson University, 258 Lehotsky Hall, Clemson, SC USA

## Abstract

**Background:**

Camera traps present a valuable tool for monitoring animals but detect species imperfectly. Occupancy models are frequently used to address this, but it is unclear what spatial scale the data represent. Although individual cameras monitor animal activity within a small target window in front of the device, many practitioners use these data to infer animal presence over larger, vaguely-defined areas. Animal movement is generally presumed to link these scales, but fine-scale heterogeneity in animal space use could disrupt this relationship.

**Methods:**

We deployed cameras at 10 m intervals across a 0.6 ha forest plot to create an unprecedentedly dense sensor array that allows us to compare animal detections at these two scales. Using time-stamped camera detections we reconstructed fine-scale movement paths of four mammal species and characterized (a) how well animal use of a single camera represented use of the surrounding plot, (b) how well cameras detected animals, and (c) how these processes affected overall detection probability, p. We used these observations to parameterize simulations that test the performance of occupancy models in realistic scenarios.

**Results:**

We document two important aspects of animal movement and how it affects sampling with passive detectors. First, animal space use is heterogeneous at the camera-trap scale, and data from a single camera may poorly represent activity in its surroundings. Second, cameras frequently (14–71%) fail to record passing animals. Our simulations show how this heterogeneity can introduce unmodeled variation into detection probability, biasing occupancy estimates for species with low p.

**Conclusions:**

Occupancy or population estimates with camera traps could be improved by increasing camera reliability to reduce missed detections, adding covariates to model heterogeneity in p, or increasing the area sampled by each camera through different sampling designs or technologies.

**Supplementary Information:**

The online version contains supplementary material available at 10.1186/s40462-021-00277-3.

## Introduction

Global environmental change has increased the need for rapid, large-scale surveys of ecological communities [38]. Such surveys are particularly important for mammals, which are often at high risk for extinction but sparsely monitored in the wild. Since most mammals are difficult to see or catch directly, passive sampling devices have become an increasingly popular alternative, particularly camera traps for studying population size or distributions of terrestrial mammals [38]. However, statistical interpretation of passive monitoring data is often challenging. Imperfect detection is a major source of error in large-scale biological surveys [42], including those based on camera trapping. Unfortunately, most mammals cannot be visually distinguished as individuals, reducing the analytical options. Instead, many practitioners use occupancy analysis to account for imperfect detection when evaluating habitat preferences or distribution [3].

Occupancy models analyze detection/non-detection data as the result of two processes: occupancy (*ψ*) is the probability of a species occurring within a spatial unit (or “site”) during the sampling season, and detection probability (*p*) is the probability that the species will be detected when it occurs at a site [22]. Replicate sites, and replicate observations at each site, are needed to estimate these parameters. In camera trap studies, practitioners typically treat individual cameras as sites and divide the data into temporal blocks (e.g. 1- or 5-day intervals) to define replicate surveys at each camera [37].

Although estimates of occupancy in this framework account for imperfect detection, it was not created for surveys in continuous habitat [5, 6]. Its suitability for camera trap data is debated due to a spatial mismatch between the fine scale measured by each camera versus the larger scale over which animals move [3, 6]. Although animals move over larger spatial areas, each individual camera monitors a small area of just a few square meters [14, 31]. Data at these two scales are linked by animal movement, but two critical details about this relationship are unclear. First, it is unknown how well the data from one camera represents its surroundings, or to what scale this inference extends [3]. Most practitioners presume their results are representative of large, vaguely-defined areas, but some studies suggest otherwise. For example Kays et al. [19] found little spatial autocorrelation in detection rates past 25 m for five Central American mammal species and Kolowski et al. [20] found no spatial autocorrelation at any scale, suggesting that cameras may represent small areas. While it might be safe to extend the inference of species presence directly in front of a camera trap to larger scales (e.g. home range size), inferring absence over this larger area is much more questionable. In this paper we consider the area immediately in front of the camera trap as the site that is occupied, and empirically evaluate the degree to which this reflects the larger (0.6 ha) plot-level occupancy.

The second issue relates to the assumption of site “closure” implicit to occupancy analysis. Occupancy models assume that surveyed locations remain either occupied or unoccupied throughout the entire sampling season, which is obviously violated in camera trap surveys since animals only briefly pass through the small area monitored by each camera [6]. To relax this, models assume that animals move in and out of sites at random, and that site use is consistent across the sampling season [21, 22]. However, this fundamentally changes the definitions of occupancy and detection probability, such that both parameters are related to animal movement at different time scales. Occupancy becomes “probability of use,” defined as the probability that an animal will use the site at least once during the study season [21, 22]. Likewise, detection probability becomes the probability of an animal passing a camera (“availability”) and the camera successfully capturing a usable photograph of the animal (“detectability”) during a replicate time period [21]. Although this concept has been described in general terms, the role of movement in the detection process has not been formalized or explored with empirical data.

In this paper we empirically describe both the detection process and the spatial representativeness of an individual camera trap using an extremely saturated array of cameras on a natural landscape. We use the camera trap data to reconstruct animal movement paths at a 10 m resolution across a plot of forest, allowing us to directly observe how representative single camera traps (10 m) are to a larger area (0.6 ha) and how animal movement and camera performance influence detection probability. This approach allows us to separate to components of detection probability into availability (was the animal in the area) and detectability (did the camera work as expected). We then simulate camera data based on these observations to investigate how well occupancy models might perform under realistic scenarios.

## Materials & methods

**Fig. 1:**
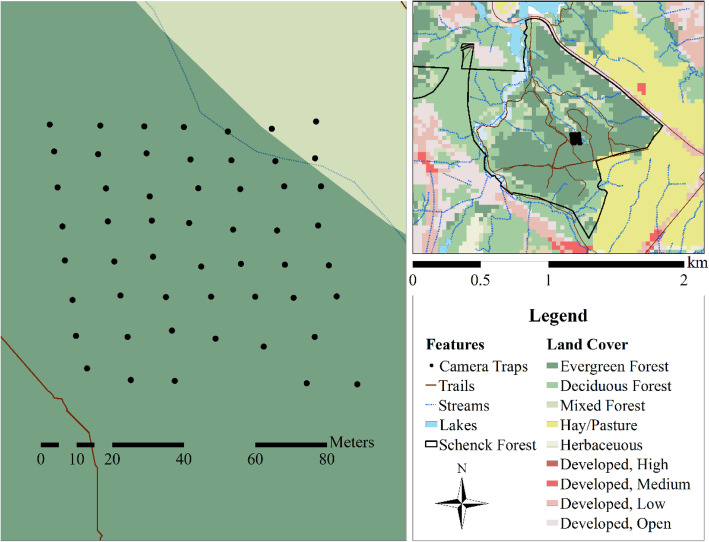
Location of fine-scale camera grid in Schenck Memorial Forest, North Carolina, USA. Camera traps were deployed at 10 m intervals, forming a 70 m by 80 m grid within a forested area. A dry creek bed ran along the northeastern corner of the grid, connecting to a larger stream off-grid

To document animal movements and camera efficacy on a natural landscape, we deployed a fine-scale array of 56 newly purchased camera traps (Bushnell Trophy Cam HD, IR flash units, 0.4 s trigger time for still photographs) within a 0.6 ha plot of loblolly pine (*Pinus taeda*) forest in Schenck Memorial Forest, North Carolina. Cameras were placed systematically at 10 m intervals, forming a 70 m by 80 m rectangular grid that we operated continuously during June 13 – July 11, 2013 (Fig. 1). The close spacing of cameras was intended to allow us to document the movement of all individuals of larger mammal species through the area, which would be expensive or impossible with animal-tracking approaches due to the difficulty of capturing and collaring 100% of a population. This setup allowed us to consider camera efficacy at the camera-level (10 m) and relate it to the larger surrounding plot (0.6 ha), although we cannot extrapolate to larger scales. The terrain of the plot was relatively flat, and the vegetation was relatively homogeneous with a relatively open understory, except for some new growth along one trail that cut through the southwest corner. We used identical settings at all cameras to ensure consistency across the grid: cameras were set on trees at knee height (0.5 m), parallel to the ground, facing north, with synchronized clocks. Each camera was set to high trigger sensitivity with no quiet period between photographs. All camera traps triggered on a person walking 10 m in front, despite the presence of understory vegetation. Each individual camera trap was considered a sample unit, and our analyses are designed to quantify their performance and how well they represent the larger area.

This grid intensively sampled animal movement providing unique insight into the process linking fine-scale animal movement to camera detections. Many practitioners would consider an area the scale of our plot (or larger) as one camera “site,” monitored by a single camera station [5]. Instead, we vastly increased survey effort, yielding over 1430 trap-nights of continuous data from one plot. Animals moving across the grid generally passed in front of several cameras, allowing us to infer movement paths. We used these reconstructed paths to assess how well the camera traps detected different mammal species.

### Movement paths

**Fig. 2:**
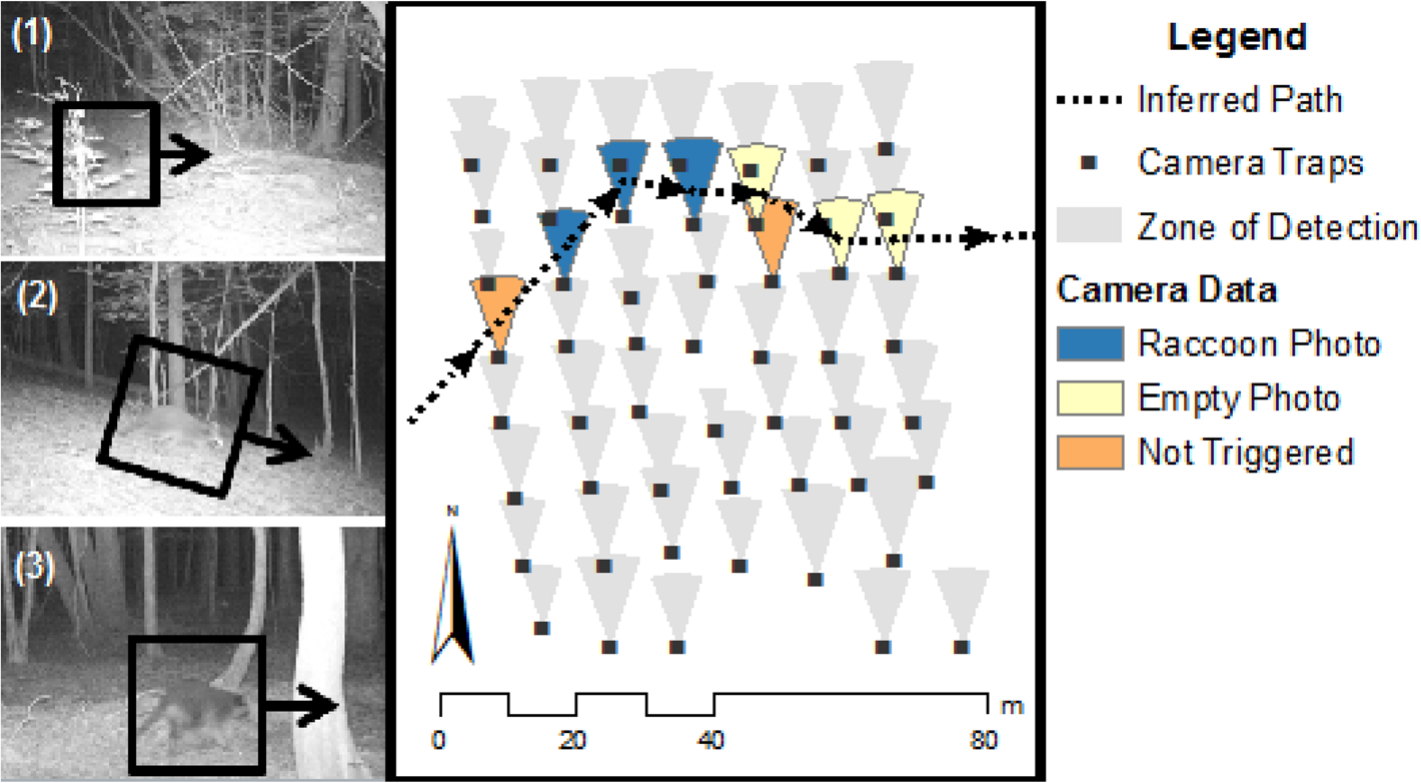
Reconstructed raccoon movement path. Three cameras photographed a raccoon as it moved east over the grid at 2:59 AM on June 26, 2013. These photos are displayed chronologically to the left of the map. Within a minute, three more cameras took empty photographs in the direction that the animal was last seen moving. The timing and location of these photographs suggest that the raccoon triggered the cameras but moved out of frame before a photograph could be taken. Inferred movement paths suggest that at least two other cameras were also visited, but not triggered. The exact location of these missed detections was inferred from the direction of travel of the animal in other pictures using a conservative rule to minimize missed detections to complete the route

We used our intensive sampling array to reconstruct fine-scale movement paths of unmarked animals moving across the grid, providing the basis for subsequent analyses (Table 1, Fig. 2). To do this, we first referenced every animal photograph to a specific place and time on the grid based on the associated camera ID and timestamp. In some cases, animals were simultaneously photographed by two camera traps. When this occurred, we only used the data from the closer camera to avoid double-counting the animal. Cameras recorded 3 consecutive photographs for each trigger, and immediately retriggered if motion was still detected. We combined consecutive images at the same camera into one ‘detection’ if they were < 60s apart.

**Table 1:** Summary of method to reconstruct movement paths and infer missed detections from a fine scale camera trap grid

1. Remove duplicates: For cases where two cameras simultaneously photographed the same animal, keep only the record from the camera closest to the animal.	
2. Consider detections close in time: Review all records < 5 min from each other if they are the same species, an empty frame, or an unidentifiable animal.	
3. Order chronologically: Mark on the map showing the time sequence of photos.	
4. Recognize direction of travel: mark on map	
5. Reconstruct movement path: Link sequential detections by drawing the shortest Euclidian distance between the detection zone where the target species was photographed, empty frames, and blurry pictures while also considering time of travel.	
6. Identify missed detections: If the shortest path between detections crossed a camera that did not record a photograph, record that as a failed detection.

We then linked associated detections together into movement paths (Fig. 2). This involved a three-step process. First, we grouped photographs into grid-level events using a simple heuristic rule chosen based on temporal autocorrelation in animal detections (Additional file 1). If photographs of the same species occurred within 5 min of one another, we grouped them together as an event that would then be examined closer in step two. We also included photographs that contained no animals (i.e., motion sensor triggered but no animal in view) or unidentifiable animals as putative missed detections if they occurred within 5 min of a known animal photograph. Next, we ordered photographs chronologically within these grid-level events using both their exact timestamps as well as the movement direction of animals within photos. This allowed us to resolve ambiguous cases where pictures occurred within the same minute or where multiple individuals visited the grid simultaneously. Finally, we constructed movement paths by connecting the shortest Euclidean distance between successive photographs. Some events only contained one detection, often occurring near edge of the grid. In these cases, we assumed the path that intercepted the fewest camera traps. While not perfect, this approach gives estimated paths of animals moving through the plot with conservative assumptions that minimize the estimated path length.

### Fine-scale space use

We used these estimated movement paths and fine-scale capture rates (photo sequences per day at individual cameras) to identify where animals moved within the grid with 10 m accuracy during three 9-day intervals. We did not formally consider the fine scale heterogeneity of the landscape on our models. However, we did a post-hoc informal consideration of the space use patterns we observed in the animals against fine-scale habitat features and animal behaviors caught on camera to explore biological reasons for these patterns. These data allowed us to describe how animal space use can vary at a fine scale and quantify how well individual camera traps represent activity in their surroundings.

### Components of the detection process

We also used the reconstructed movement paths to assess the relative effects of animal movement (“availability”) and sensor efficacy on detection probability. We conceptualized detection as a four-step process. For a camera to photograph an individual animal, the animal must (1) enter the general area (in this case the plot) and subsequently (2) pass in front of the camera. The animal must then (3) activate the camera’s passive infrared (PIR) motion trigger and (4) remain in frame long enough for the camera to capture the animal in a picture.

This can be formalized using a modification of the Royle-Nichols model for abundance-induced heterogeneity in detection probability [33]:

1 $$ {p}_i=1-{\left[1-\left({r}_c\ast {r}_t\ast {r}_p\right)\right]}^{N_i} $$

Here, *p*_*i*_ is the probability of one camera detecting the target species on day *i*, and *N*_*i*_ is the number of times individual animals entered the surrounding area (here, represented by the 0.6 ha plot) on day *i*. We stress that the meaning of parameters here differ slightly from the original equation. Particularly, *N*_*i*_ in this case describes the daily number of visits to the plot, not necessarily the number of individual animals since the same individual could revisit the plot. The remaining variables are a set of conditional probabilities:

2 $$ {r}_c=\Pr \left(\mathrm{animal}\ \mathrm{passes}\ \mathrm{the}\ \mathrm{camera}\ |\ \mathrm{animal}\ \mathrm{on}\ \mathrm{the}\ \mathrm{grid}\right) $$

3 $$ {r}_t=\Pr \left(\mathrm{animal}\ \mathrm{triggers}\ \mathrm{the}\ \mathrm{camera}\ |\ \mathrm{animal}\ \mathrm{on}\ \mathrm{the}\ \mathrm{grid},\mathrm{passes}\ \mathrm{in}\ \mathrm{front}\ \mathrm{of}\ \mathrm{camera}\right) $$

4 $$ {r}_p=\Pr \left(\mathrm{animal}\ \mathrm{is}\ \mathrm{photographed}\ |\ \mathrm{animal}\ \mathrm{on}\ \mathrm{the}\ \mathrm{grid},\mathrm{passes}\ \mathrm{camera},\mathrm{triggers}\ \mathrm{camera}\right) $$

Our choice to separate *N*_*i*_ and *r*_*c*_ into two components is an arbitrary decision, which allowed us to conceptualize space use on two spatial scales. Many practitioners assume that space use at neighboring camera locations is homogeneous and describe animal movement at a camera trap interchangeably with movement in its immediate surroundings (represented here by the camera and the grid) [5]. By separating *N*_*i*_ and *r*_*c*_, we were able to investigate the suitability of this assumption. It would be equally valid to describe the detection process solely in terms of the area in front of the camera, holding *r*_*c*_ equal to 1 and redefining *N*_*i*_ as the daily number of visits to the camera itself.

Using this framework, we empirically estimated individual components of the detection process (*N*_*i*_, *r*_*c*_*, r*_*t*_*, r*_*p*_) for each species as described in Table 2. To facilitate interpretation, we averaged daily number of visits to the grid (*N*_*i*_) across days and probability of passing a camera (*r*_*c*_) across all cameras. For the average visit rate ($\overline{N}$), we averaged the number of movement paths recorded for each species across days. For the average probability of an animal passing an individual camera given a visit (${\overline{r}}_c$), we calculated the average proportion of cameras that were passed per movement path, whether or not the camera took a picture. Similarly, we calculated probability of a camera triggering given a pass (*r*_*t*_) as the proportion of all camera visits that resulted in a photograph, regardless of whether the animal was captured in the image. Lastly, we calculated probability of a camera capturing a photograph of an animal that was identifiable to species given its trigger (*r*_*p*_) as the proportion of animal-caused triggers that resulted in a photograph of the animal.

**Table 2:** Definitions for components of detectability and how they were estimated. We formalized daily detection probability as a four-step process involving animal movement and camera efficacy. The probability of a camera detecting the target species involves the number of times per day that animals visit the plot ($\overline{N}$), the probability that these animals will subsequently pass an individual camera (${\overline{r}}_c$), and the probability of the camera sensing the animal and triggering fast enough to take an identifiable picture (*r*_*t*_, *r*_*p*_)

Parameter	Statistical Description	Estimator Definition
$\overline{N}$	Average visit rate	Mean number of paths crossing the grid per day
${\overline{r}}_c$	Pr(Pass | Visit)	Probability that animal on grid passes a camera
*r*_*t*_	Pr(Trigger | Visit, Pass)	Proportion of passes resulting in any photograph
*r*_*p*_	Pr(Photo | Visit, Pass, Trigger)	Proportion of photographs that contain the animal

We estimated average daily detection probability ($\overline{p}$) for each species by adding the observed components of detection into Eq. 1. Since *N*_*i*_ is an integer count, we could not directly insert visit rate $\overline{N}$ into the equation. Instead, we generated 10,000 random values of *N*_*i*_ from the Poisson distribution $Pois\left(\overline{N}\right)$, calculated *p*_*i*_ for each sample, and averaged across these simulated values to calculate $\overline{p}$. To explore the role of camera performance in detection probability, we also calculated $\overline{p}$ under a hypothetical scenario in which cameras perfectly detected passing animals (*r*_*t*_ = *r*_*p*_ = 1.0), which we denote ${\overline{p}}_{ideal}$.

### Simulation

To evaluate the importance of our empirical results about unmodeled detection heterogeneity on occupancy estimates we simulated data to explore several realistic camera trapping scenarios. We make no assumptions about the size of area measured by the locations in this simulation but tested for the effect of heterogeneity of detection process on occupancy estimates. We considered three levels of occupancy probability (*ψ* = 0.3, 0.5, 0.7) and four levels of average daily detection probability ($\overline{p}=0.05,0.10,0.20,0.30,0.50$). We simulated unmodeled heterogeneity by drawing camera-specific detection probability from a beta distribution, parameterized to have a standard deviation of 0.001,0.1,0.05,0.075 and 0.1. For each species, we simulated detection probabilities generated from beta distributions representing each combination of $\overline{p}$ and sd (i.e., 20 different distributions each; Supplementary Figure 1). For each combination, we simulated 500 datasets in Program R [29] and estimated occupancy probability using single-species, single-season occupancy models in R-package ‘unmarked’ [8]. Each simulation included 100 camera locations, each sampled for 20 days. Since many practitioners bin their data into blocks (e.g., 5-day replicates) to increase detection probability, we analyzed each scenario as 1- and 5-day blocks.

We considered two measurements for model accuracy: bias and error rate. We defined bias as the average difference between true occupancy probability (*ψ*) and estimated occupancy probability ($\hat{\psi}$). Similarly, we defined error rate as the proportion of estimated 95% confidence intervals (CIs) that failed to overlap with the true value. Error rate should be 0.05 if CIs are estimated accurately.

## Results

Fifty-three out of 56 cameras ran continuously over a month, yielding over 1430 trap-nights of data. Three cameras malfunctioned during the sampling period and were therefore excluded from the analysis. Camera traps detected eight species of terrestrial mammals, including humans and small rodents, with photographs of white-tailed deer (*Odocoileus virginianus*, hereafter ‘deer’) occurring most frequently (1408 detections, 21 paths). We focused on deer and three additional species: northern raccoons (*Procyon lotor*, hereafter ‘raccoon’, 18 paths, 36 detections, 10 inferred detections), coyote (*Canis latrans,* 14 paths, 27 detections, 7 inferred detections), and gray fox (*Urocyon cinereoargenteus*, 4 paths, 7 detections, 3 inferred detections). We also had 145 detections of Eastern gray squirrel (*Sciurus carolinensis*) and 78 detections of Virginia opossum (*Didelphis virginiana*, hereafter ‘opossum’), for a total of 1702 animal detections.

We had 211 photographs containing “No Animal”, most of which were associated with animal activity, as 57% (*n* = 120) occurred within 5 min of an animal photograph somewhere on the grid. This is much higher than one might expect due to chance, as only 5% of the 43,200 min in our total 30d sample fell within 5 min of an animal detection. Furthermore, these “No Animal” images were also confirmed as animal-triggered photographs based on pictures of animals moving in the direction of the ‘No Animal’ camera moments before these detections. The remaining “No Animal” photographs were probably explained by blowing vegetation heated by the sun, or animals moving through the edge of our plot, as they occurred mostly during the day (when sun is likely to heat vegetation and cause it to trigger the passive infrared motion sensor [2] and twice as frequently along the outermost edges of the grid.

### Movement paths

We focused our analyses on four of the most common species: deer, raccoons, coyotes, and gray foxes. We analyzed movement paths for a 10-day subset of data for deer due to a high volume of detections but used the full dataset for the other three species. Squirrels and opossums were also detected but excluded from our path analysis because their detections were too spatially and temporally disjointed to reliably infer movement paths. This might be due to the semi-arboreal nature of these species, which could allow them to traverse the grid above the cameras’ line of sight, or their smaller body size, which would decrease the area over which they triggered cameras, leaving some areas within our plot where we would be less likely to detect them [32].

### Fine-scale space use

**Fig. 3:**
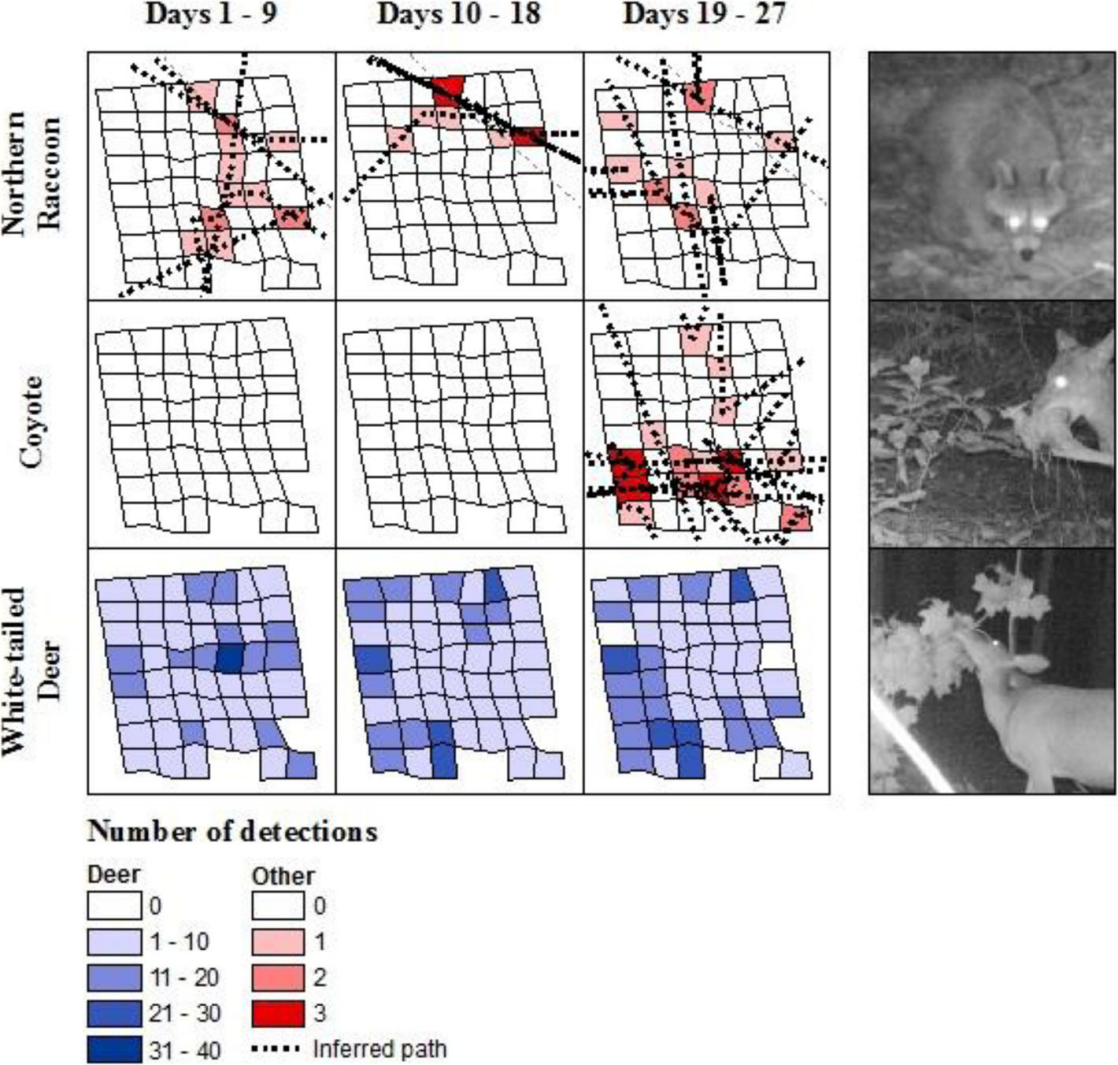
Spatiotemporal variation in animal detections on the fine-scale camera grid. Uneven distribution of animal detections within the grid reflects fine-scale variation in animal space use. We were often able to infer likely biological reasons for this variation from behaviors observed in the photos. Some raccoon paths coincided with a small dry creek bed. Deer activity briefly increased at the center of the grid on days 5–7 when a live tree branch fell from the canopy. Coyote activity dramatically increased during the last few days. Individuals were seen carrying food, suggesting that this increase was related to a feeding event. Deer movement paths are not shown because there were too many to visualize clearly

**Fig. 4:**
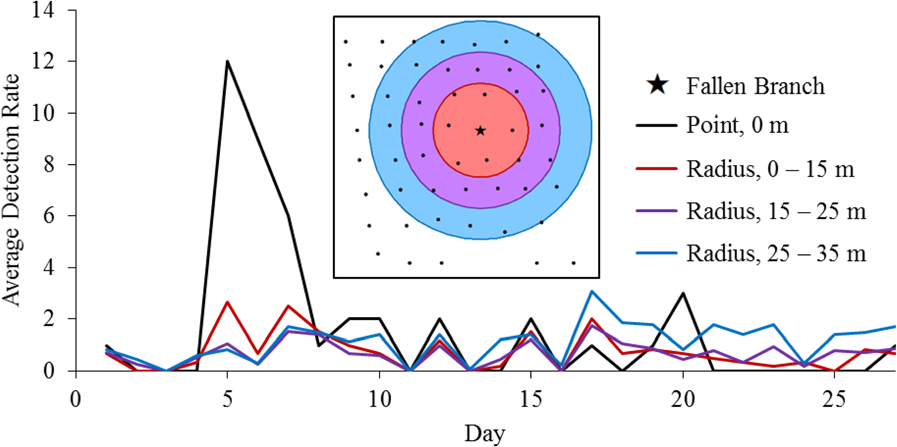
The graph shows deer detection rates by camera traps set at increasing distances from a fallen tulip-poplar branch. The inset map shows a bullseye of detection radii over the grid of cameras (black dots, separated by 10 m). The branch fell on day 5, and detection rates remained high for the next 2 days until all leaves on the branch were consumed. No discernable increase in deer detections was observed at any adjacent cameras

**Fig. 5:**
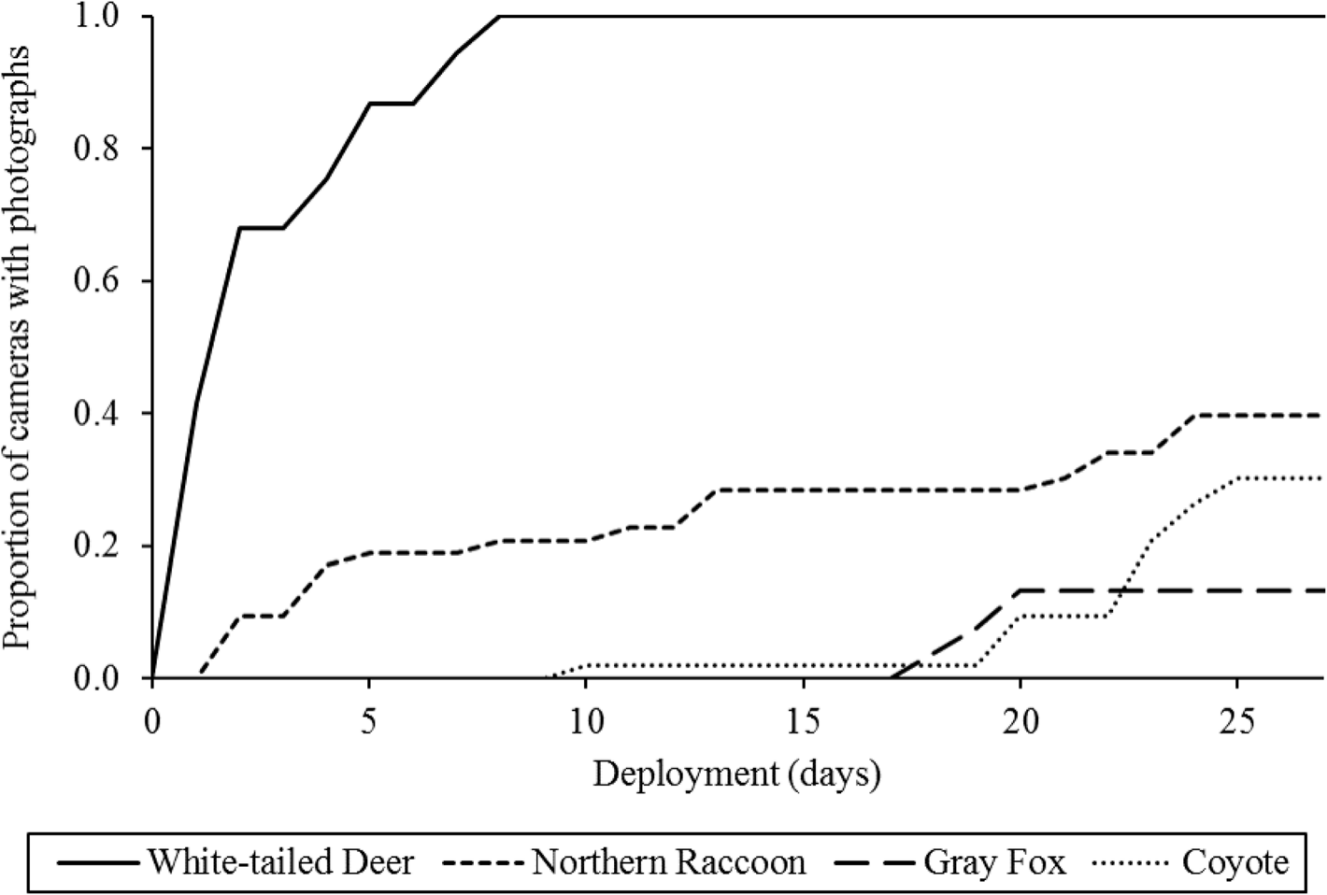
Proportion of cameras 53 cameras in one 0.6 ha plot with detections of four species over 1 month of sampling. Coyotes and gray fox visits accumulated quickly during short bursts of activity towards the end of the survey, while deer and raccoons were steadily present throughout the period

We found that animals used the fine-scale plot unevenly in both space and time. In some cases, this variation aligned with species-specific foraging behaviors recorded in the camera trap pictures (Fig. 3). For example, capture rates of raccoons were relatively high along a dry creek bed leading to a small stream north of the grid (Fig. 1). Likewise, deer activity was highly concentrated at a single station in the center of the grid during the first 9 days of the study. Camera trap photos revealed that a tulip-poplar (*Liriodendron tulipifera*) branch had fallen from the canopy during a storm, providing the deer with a rich but short-lived foraging opportunity (Fig. 4). Deer used the grid more evenly after this resource had been consumed, and detection was not obviously worse in the more heavily vegetated southwest corner of the plot. Coyote activity was extremely clustered in both space and time, erupting in a series of east-west oriented paths on the south half of the grid for a few days towards the end of the study (Fig. 5). Camera trap pictures showed individuals scent marking and carrying food items from an unseen location east of the grid, presumably related to a hunting or scavenging event. Foxes were also first detected during this same time, possibly as scavengers on the coyote kill. In total, deer were present for the entire study and eventually were detected by all cameras, raccoons were nearly always detected but only by a subset of cameras, while foxes and coyotes were only detected towards the end of the month, and only by a small subset of cameras (Fig. 5).

### Components of the detection process

We used movement paths of the four focal species to investigate how reliably cameras photographed passing animals (Table 3). Generally, the cameras photographed passing animals more frequently than they failed to trigger or triggered late (i.e., producing “empty” images with no animals). The proportion of passes successfully documented with a camera trap photograph varied among species, from just 29% in gray foxes to 86% in deer. However, trigger failures (“no photos”) were always more common than failures due to rapid animal movement (“empty photos”).

**Table 3:** Raw observed outcomes of animals passing within 10 m of camera traps. These observations were used to estimate the components of detectability described in Table 3. Camera outcomes are reported as the average proportion of camera visits that result in identifiable animal photographs, empty or unidentifiable photographs, or no photographs (no camera trigger). These movement paths are from a 10-day subset of data for deer due to a high volume of detections but we used the full dataset for the other three species

Species	Proportion of Photo Outcomes			Sample Sizes	
	***Identifiable***	***Empty or Unclear***	***No Photos***	***Camera passes***	***Paths***
*Deer*	0.86	0.05	0.09	123	21
*Raccoon*	0.47	0.13	0.39	76	18
*Coyote*	0.57	0.15	0.28	47	14
*Gray Fox*	0.29	0.12	0.58	24	4

Restructuring these values into components of detectability provided further insight into the detection process (Table 4). The probability of an animal encountering a camera (*r*_*c*_) was consistently the smallest component of detectability, varying between about 0.06 and 0.11 for our focal species. This suggests that the probability of an individual camera trap encountering an animal on the plot was low on average, such that a single randomly placed camera trap may poorly represent animal activity within even modestly larger areas (i.e., our 0.6 ha plot). The clustering of animal movement paths into corridors and hotspots furthermore shows that this encounter probability varies substantially across short distances.

**Table 4:** Estimated components of detectability. Components of detectability are defined as follows: $\overline{N}$ is the average number of movement paths crossing the plot per day over a 26.8d period; ${\overline{r}}_c$ is the average probability of an animal visiting a given camera during a plot visit; *r*_*t*_ is the probability that a camera triggers, given a camera visit; and *r*_*p*_ is the probability of a camera producing a useable photograph of the animal if the camera triggers. Average daily detection probability ($\overline{p}$) and ideal average daily detection probability (${\overline{p}}_{ideal}$) for a single camera are empirically derived from these components, with ${\overline{p}}_{ideal}$ representing a hypothetical scenario in which cameras perfectly detected passing animals

***Species***	$\overline{\boldsymbol{N}}$	${\overline{\boldsymbol{r}}}_{\boldsymbol{c}}$	***r***_***t***_	***r***_***p***_	$\overline{\boldsymbol{p}}$	${\overline{\boldsymbol{p}}}_{\boldsymbol{ideal}}$
*Deer*	0.78	0.11	0.91	0.95	0.149	0.170
*Raccoon*	0.67	0.08	0.61	0.78	0.025	0.052
*Coyote*	0.52	0.06	0.72	0.79	0.018	0.031
*Gray Fox*	0.15	0.11	0.42	0.70	0.005	0.016

**Fig. 6:**
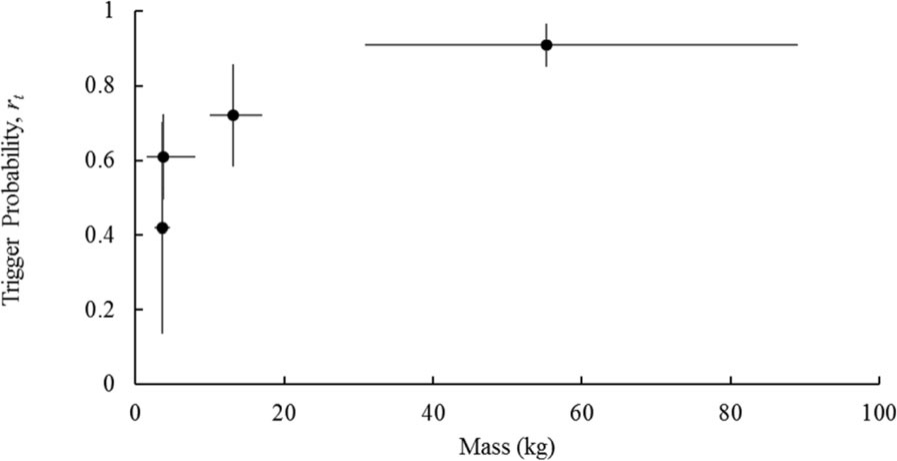
Relationship between trigger probability and body mass for four focal species (ascending order by weight: gray fox, raccoon, coyote, white-tailed deer). Error bars show standard deviation. Body mass values come from North Carolina animals in the mammal collections of the NC Museum of Natural Sciences

Trigger probability (*r*_*t*_) and photo probability (*r*_*p*_) were generally large compared to encounter probability (*r*_*p*_) and varied among species. Trigger probability showed the most between-species variation, apparently increasing with body mass (Fig. 6).

### Simulations

**Fig. 7:**
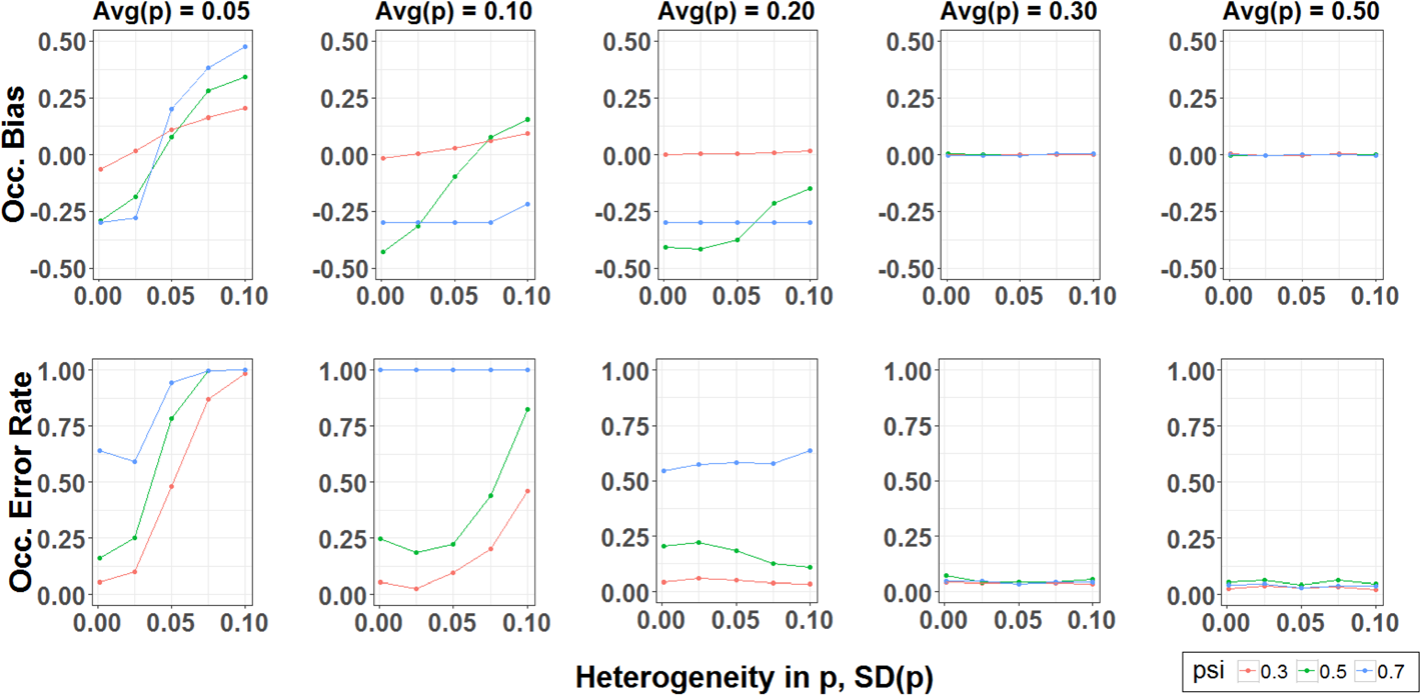
Effects of heterogeneous detection probability on occupancy estimates. Each scenario was evaluated by simulating *n* = 500 datasets in which 100 camera traps were deployed 20 days and analyzing the data with the R-package ‘unmarked,’ treating each day as a replicate “visit.” We observed similar trends when analyzing the data as four 5-day intervals. Occupancy bias is defined as the average difference between the point estimate for occupancy probability ($\hat{\psi}$) and true occupancy probability (*ψ*). Occupancy error rate is defined as the proportion of simulations in which the estimated 95% confidence interval for $\hat{\left(\psi \right)}$ did not overlap with the true value

The robustness of occupancy models to unmodeled heterogeneity in *p* depended on the average detection probability across cameras, $\overline{p}$ (Fig. 7). When average detection probability was low ($\overline{p}$ < 0.3), variability in p adversely affected occupancy estimates. The 95% confidence intervals associated with $\hat{\psi}$ became less reliable as variability in *p* increased for detectability lower than 0.1, and bias in occupancy point estimates shifted from negative to positive in some cases. Binning the data into 5-day intervals improved parameter estimation in some cases, but these improvements were only substantial when $\overline{p}$ > 0.2, relatively high compared to our empirical observations. Conversely, unmodeled heterogeneity in *p* did not affect occupancy estimates when average detection probability was high ($\overline{p}$ ≥ 0.3). In these cases, occupancy point estimates were unbiased and 95% confidence intervals were accurately specified.

## Discussion

Occupancy modeling is one of the most common analytical methods for documenting habitat choice and distribution from camera trap studies, but questions remain about the size of the area represented by each camera, how reliably cameras photograph passing animals, and how well occupancy models describe these processes. We addressed these questions using a unique fine scale (10 m) measure of animal space use. Our grid of cameras allowed us to estimate the movement of practically all individuals of multiple species using a given area for the first time, providing insight into how representative one camera-site is of the surrounding area (within 0.6 ha) and how reliably cameras photographed animals when they pass by (within 10 m). Animal space use was highly variable in both space and time, such that single cameras are not likely to represent the animal activity of the surrounding area well for most species. Camera performance in detecting passing animals was also poor, and animals frequently failed to trigger the camera or moved out of frame before the image was taken; this was particularly true for smaller, faster moving species. Collectively, non-random space use and low camera performance can result in low, variable detection probabilities, which can in turn bias occupancy estimates. However, our simulations suggest that modestly increasing detection probability could offset this issue.

Cameras frequently missed passing animals, either by failing to trigger, or triggering too slowly (*r*_*t*_ and *r*_*p*_, Table 4). These results are similar to those reported by studies using time-lapse images to recognize missed detections in Reconyx brand camera traps [12, 39] or CCTV to compare detections of otters [7]. Trigger probability (*r*_*t*_) appeared to increase with the body mass of the target species (Fig. 6), and the occurrence of “blank” images in which mammals moved out of the frame before the camera took a photograph suggests that cameras may miss fast-moving animals. These results reflect the imperfect sensitivity of the PIR sensors that trigger camera taps, requiring a threshold of change in the thermal signature of the trigger area that might not be met by small animals, larger animals further away, or animals moving very fast [24, 32]. In addition, although each camera triggered on a human out to 10 m, it is probable that our smaller-bodied or faster moving target species would not be detected perfectly at the far-end of our detection area. Thus, our results on detectability are specific to the 10 m detection area defined by our sampling grid but are broadly relevant because they emphasize the point that camera traps do not get a perfect measure of animal movement. We also note that the PIR motion sensor triggers on changes to the infrared signature in front of the camera, typically a warm blooded animal moving across cooler background vegetation [40]. Given our work was done during the warm season, when temperatures sometimes approached body-temperature for many mammals, the sensors might have been less sensitive than if run in cooler environments, where there is more contrast between animal and background [13].

The fine-scale variation in animal availability is more difficult to control for in models if the important fine-scale features of the landscape are not known and measured ahead of time and can substantially depress overall detection probability. The probability of an animal passing a given camera when it visited the plot (*r*_*c*_) was very low for all focal species in our study, ranging between 0.06 and 0.11. This indicates that a single, randomly placed camera trap would have likely missed most animal movement in the surrounding area during our one-month sample, and emphasizes the importance of maximizing the number of locations sampled by a study design [20, 35]. In some cases, animal movement is focused around fine-scale habitat features, such as movement corridors and localized foraging resources, and these differed among species. These patterns were more pronounced in some species than others. For example, deer activity temporarily spiked at one location due to high-quality foraging opportunities, but activity elsewhere on the plot was more uniform. Conversely, coyote activity was concentrated within a heavily used fine-scale movement corridor, and generally absent elsewhere on the grid. Targeting these features when setting the cameras would be difficult to do in a consistent way, and might not help as a recent evaluation found no benefit for targeting game-trails for detecting most species [9]. The lack of detections for coyotes and foxes at the start of the study (Fig. 5) could have also been due to neophobia directed at the sudden appearance of so many camera traps [23].

In occupancy studies, this variation equates to fine-scale heterogeneity in animals’ availability for sampling, thus introducing unmodeled heterogeneity in detection probability across camera locations. This is extremely important to consider in camera trap occupancy surveys, as unmodeled heterogeneity violates model assumptions and can lead to biased parameter estimates. We used simulations to investigate how severely these violations might affect occupancy models and found them only problematic when average detection probability was low across cameras ($\overline{p}<0.3$). In these cases, occupancy estimates were biased and had inaccurate confidence intervals. Thus, this problem would be worse with rare species, which are often the target of faunal surveys. We should point out that our simulations presume unmodelled heterogeneity in *p* is randomly distributed across sites, and are thus conservative in estimating bias comparison to situations where *p* would be associated with some other unmodeled habitat factors, or variable camera models.

Imperfect trigger and photo probabilities (*r*_*t*_, *r*_*p*_ < 1) are not intrinsically problematic for occupancy models, but they may affect parameter estimation by reducing camera-specific detection probabilities, which are already small and variable due to fine-scale animal movement (Table 4). By further reducing average *p*, imperfect *r*_*t*_ and *r*_*p*_ increase the bias in occupancy estimates (Fig. 7). The most obvious improvements needed are faster trigger times to reduce missed photos and increasing sensor sensitivity to detect smaller animals further away. However, the benefits from these upgrades might be relatively modest. Based on the components of detection that we estimated, daily detection probabilities for all focal species would still be low ($\overline{p}$ < 0.2) if cameras detected passing animals perfectly (Table 4). Thus, technological improvements to camera speed or sensitivity are not likely to sufficiently increase $\overline{p}$ unless the probability of encountering the species (*r*_*c*_ and *N*) is also increased.

Some common approaches for increasing the likelihood of encountering animals that use an area are to bait the camera, place it along habitat features that are frequently used by the target species (e.g., game trails), or deploy cameras for a longer period of time. While these approaches may be appropriate for capture-recapture studies, they are less suitable for occupancy studies where individual cameras are treated as sites. Baiting cameras may be the most problematic of the three, as the area from which they attract animals is unknown and variable depending on weather, effectively sampling plots of unknown, variable sizes [6]. Furthermore, attraction to bait can introduce heterogeneity due to behavioral variation among individual animals within [11] and between species [1, 25] and temporal variation in detection probability as the bait degrades or is eaten. Nonetheless, these problems might be offset by increased detection probability and by keeping an animal in front of the camera long enough for a good picture, especially for single species studies where comparisons across the community are less important [4]. Placing camera traps along fine-scale habitat features (e.g. roads, trails, logs) is another common practice and may be more suitable for occupancy studies [20]. This approach still requires representative sampling of the landscape, and can also be put in context if combined with truly random, off-trail, sites [18]. Extending deployment length may be the most innocuous of these three common solutions, but it may be ineffective, particularly if species have very low detection rates [17, 34, 35], or simply never use a particular site due to fine-scale habitat factors (Fig. 5). Furthermore, long deployments can violate the assumption of population closure if they run long enough to capture immigration/emigration dynamics, which could be a problem for some types of analyses. Finally, setting cameras to also take time-lapse images to complement motion-triggered pictures could also help quantify the detectability of species in cases where the time-lapse image recorded an animal but the motion sensor didn’t trigger [12, 39].

Alternatively, suitable increases in detection probability might be achieved by both increasing camera performance and increasing the true spatial extent of area monitored. One promising technological approach would be to develop 360° camera traps. Indeed, a 360° camera would both survey a larger area and also be virtually immune to the problem of losing view of animals out of the frame from slow trigger times, improving all aspects of the detection process. Sampling designs based on camera arrays might also offer a solution. For example, a number of studies have shown that placing multiple cameras at the same point can increase detection probabilities for most species [27, 41]. The downside of this approach is that the increased effort and costs needed to sample one site would likely result in fewer sites sampled overall [10].

Both of these solutions offer important improvements over current camera trap practices, but they also require additional research. Many camera trap researchers are interested in studying animal space use at much larger spatial scales than those that are conventionally measured [38], and both solutions would provide larger, more clearly-defined sites. However, unmodeled variation in detection probability would still occur in both of these scenarios. Average detection probability would likely be large enough to offset potential biases in occupancy estimates, but additional research to determine whether such variation might affect model selection is needed.

Statistical issues due to variability in *p* might also be ameliorated within the modeling framework by including fine-scale habitat covariates on detection probability or including camera-specific random effects on detection probability. We encourage additional research to determine the extent to which fine-scale habitat covariates can account for this heterogeneity and point out that the variation observed within our forest plot occurred across distances as little as 10 m, below the resolution of most remotely sensed data. This highlights the importance of field-recorded habitat covariates collected when setting cameras, especially those related to the most elusive species (i.e., lowest detection probability).

Many camera trap researchers may also be interested in tangential questions regarding how closely they might space their cameras or how densely they should sample their study area. These are important design questions, but we refrain from making specific recommendations based on our data because they are all at relatively fine scale. We also caution that our observations of fine-scale heterogeneity do not justify close spacing of camera traps, as autocorrelation can still occur in the presence of fine-scale heterogeneity. Indeed, model based estimates from another study in our region suggest that occupancy is significantly autocorrelated up to 0.4 km for red foxes (*Vulpes vulpes*) and 0.8 km for coyotes [30]. We instead suggest that practitioners follow current best practices and strive to sample spatial locations that are truly representative of their study areas. Excessively close camera placement (e.g., 10-20 m) may be inefficient, but autocorrelation seems unimportant at these scales [20], and can also be addressed using newly-developed occupancy models that account for complex spatial structures in occupancy and detection probability (e.g., [15, 26, 30]).

Our research is the first of its kind to estimate the movement of all individual animals from multiple species across a (small) natural landscape using camera traps. However, we recognize that our approach was not perfect, and relies on certain assumptions that undoubtably introduced uncertainty into our estimates. First, we presumed that these species should be detected to 10 m away (the size of our grid), but detectability is likely to decrease with distance even at this scale, so that some of the non-triggers could have been animals walking along the far edge of a grid cell and not triggering the camera. Second, we considered animal detections within the plot < 5 min apart as one animal while they might have been two different individuals. We were able to account for this in many cases when a group of animals could be seen in the photographs, or by accounting for exact time stamps and direction of travel, but might have missed a few occasions. We presumed that ‘No Animal’ photographs near an animal detection in space and time were a missed detection of that species, which might not always be the case, although blank photographs were much more likely near known animal deteections, suggesting this was a primary cause. When infering missed detections, we presumed an animal walked a straight line between known detections, which might have underestimated missed detections of nonlinear movement. Additionally, movements were difficult to map along the edge of the grid, and could have been inaccurate for cases where raccoons or gray foxes ascended or descended into a tree to rest (although we never observed such tree climbing in the pictures). Nonetheless, we feel that these problems are minor compared to the level of insight provided by such complete sampling of the movement of the animal community, and encourage others to extend this work through improved study designs and image processing.

## Conclusions

Overall, we observed pronounced mismatch between the small spatial scale recorded by individual camera traps versus larger spatial scales of interest to practitioners for ecological inferences, such that absence at a given camera does not reliably indicate absence from the immediately surrounding landscape. Additionally, by dissecting the two components of detectability (availability at the site and probability that the camera triggers when the animal is there) we hope to highlight that occupancy analyses with camera trap data violates the assumption of spatial closure, and thus should properly be interpreted as “probability of use” [21, 22]. We recommend that wildlife ecologists pursue improved sampling designs to increase site size and detection probability and adopt existing models that better account for heterogeneity and autocorrelation structures due to fine-scale animal movement. We suggest that the development of 360° panoramic camera traps or survey designs using nested camera trap arrays offer two potential solutions for improving occupancy surveys. We also encourage additional research into the detectability of different species-camera combinations, and between small and large-scale movement through multiple-camera designs [16, 27, 28] and combined camera-animal tracking studies [36].

## Supplementary Information

**Additional file 1.** Fine-scale temporal correlograms for animal detections in Schenck Forest camera grid.

**Additional file 2: **Distributions used to simulate unmodeled heterogeneity in detection probability for occupancy models. We simulated five levels of average daily detection probability (=0.05,0.10,0.20,0.30 and 0.50) and five levels of detection probability variation (sd = 0.001,0.1,0.05,0.075 and 0.1). For each species, we simulated camera-specific detection probabilities by pulling from beta distributions representing each combination of and sd (i.e., 20 different distributions each). Probability density functions for each combination of and sd are given, along with their a and b parameter values.

**Additional file 3.** Camera trap data.

## Data Availability

The raw data are available as a supplement to this article (Additional file 3).
