# Supplementary material for: Empirical evaluation of the spatial scale and detection process of camera trap surveys
Source: Mov Ecol. 2021 Aug 14;9:41. doi: 10.1186/s40462-021-00277-3 (PMC8364038; doi:10.1186/s40462-021-00277-3)
Supplement: Supplementary file 1 — Additional file 1. Fine-scale temporal correlograms for animal detections in Schenck Forest camera grid. [file 40462_2021_277_MOESM1_ESM.docx]

**Appendix A**

*Fine-scale temporal correlograms for animal detections in Schenck Forest camera grid*

Prior to analysis, we used the JMP Pro 12 Time Series Analysis platform (SAS, 2015) to study temporal autocorrelation and partial autocorrelation in animal detections. The goal of this study was to see at what time scale detections were autocorrelated on the grid to inform how long individuals tended to spend on the study site. We analyzed the detections two ways. First, we considered autocorrelation between any animal detections, regardless of species. Second, we considered autocorrelation between detections within individual species with at least n=30 photo sequences. In both cases, we collapsed data into minute-by-minute detection histories, where ‘1’ denotes detection anywhere on the grid and ‘0’ denotes no detections on the grid for a given minute. We considered different time lags for the two scenarios. When fitting the multispecies correlograms, we considered a maximum time lag of 60 min and a forecasting period of 25 min. When fitting the single-species correlograms, we reduced the maximum time lag to 30 min. All other settings remained the same. We judged temporal autocorrelation to be insignificant when it consistently dropped within ±2 standard deviations of 0.

Temporal autocorrelation of all species combined persisted up to 40 minutes, but autocorrelation for individual species dissipated more quickly. Autocorrelation dissipated within 5 minutes for gray squirrels, raccoons, and coyotes, and dissipated within 20 minutes and 26 minutes for opossums and white-tailed deer, respectively. We selected to use a 5-minute grouping rule since it is more conservative and reduces the chances of grouping unrelated photographs.

Table A1. Results of time series analyses on animal detections.

| **Species** | **Max. Time Lag (min)** | **Forecasting (min)** | **Threshold (min)** |
| --- | --- | --- | --- |
| All (combined) | 60 | 25 | 40 |
| White-tailed deer | 30 | 25 | 26 |
| Eastern gray squirrel | 30 | 25 | 3 |
| Virginia opossum | 30 | 25 | 20 |
| Northern raccoon | 30 | 25 | 5 |
| Coyote | 30 | 25 | 5 |
