# Supplementary figures and images for: Empirical evaluation of the spatial scale and detection process of camera trap surveys
Source: Mov Ecol. 2021 Aug 14;9:41. doi: 10.1186/s40462-021-00277-3 (PMC8364038; doi:10.1186/s40462-021-00277-3)

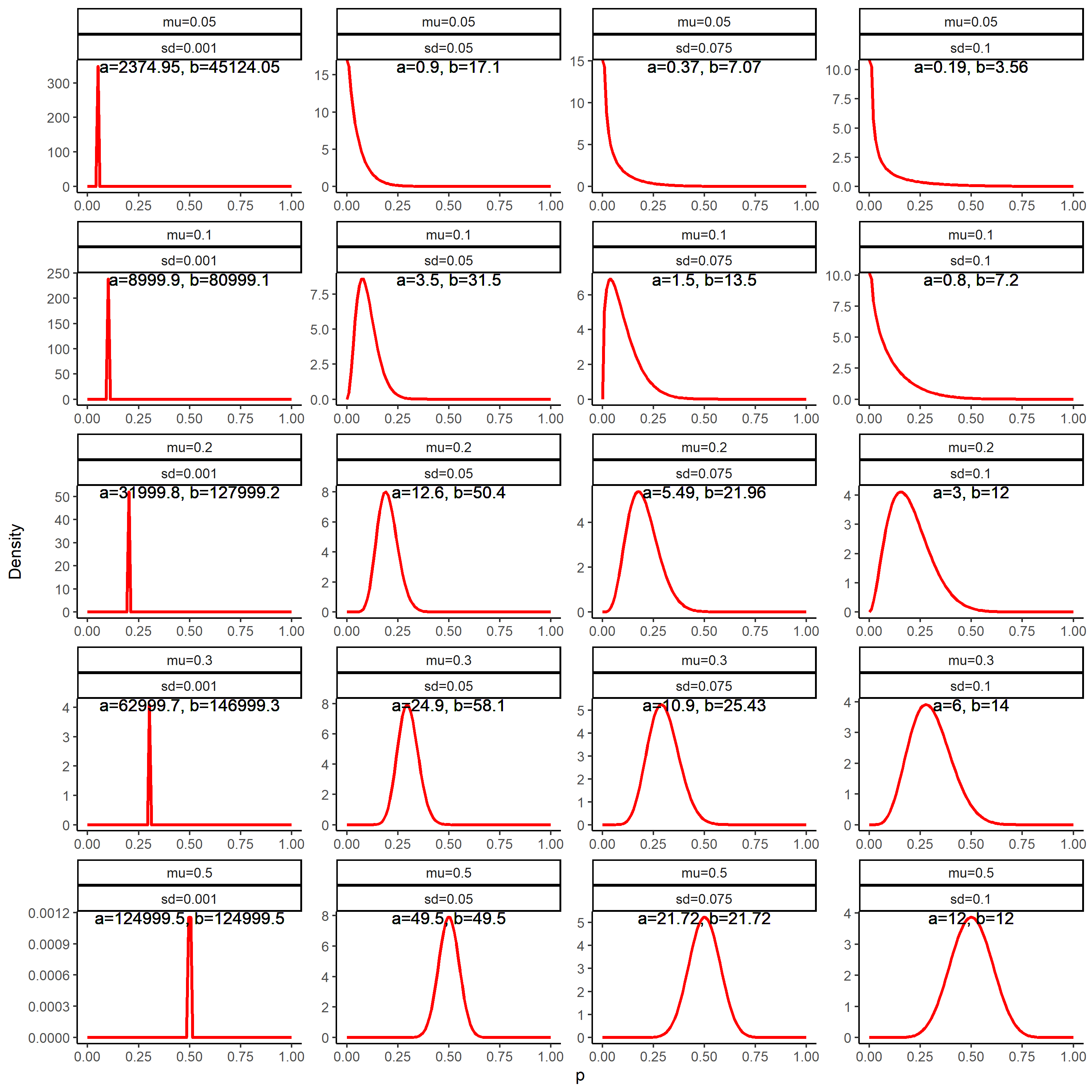

Supplement: Supplementary file 2 — Additional file 2: Distributions used to simulate unmodeled heterogeneity in detection probability for occupancy models. We simulated five levels of average daily detection probability (=0.05,0.10,0.20,0.30 and 0.50) and five levels of detection probability variation (sd = 0.001,0.1,0.05,0.075 and 0.1). For each species, we simulated camera-specific detection probabilities by pulling from beta distributions representing each combination of and sd (i.e., 20 different distributions each). Probability density functions for each combination of and sd are given, along with their a and b parameter values. [file 40462_2021_277_MOESM2_ESM.tiff]
